# Exploring the Frontiers of Health Tourism: A Bibliometric Analysis of Research Themes and Trends

**DOI:** 10.7759/cureus.66832

**Published:** 2024-08-14

**Authors:** Alan Lukose, Sajan N Thomas, Shaiju KS, Jacob Bose, Gibin Jacob, Bobby Simon

**Affiliations:** 1 Department of Commerce, Prajyoti Niketan College, Pudukad, Thrissur, IND; 2 Department of Hospitality and Tourism, Marian College Kuttikkanam Autonomous, Kuttikkanam, IND; 3 Department of Commerce, St. Paul’s College Kalamassery, Kalamassery, IND; 4 Department of Commerce, St. Thomas College, Pala, Palai, IND

**Keywords:** citespace, biblioshiny, bibliometric analyses, wellness tourism, medical tourism, health tourism

## Abstract

Health tourism, encompassing both wellness and medical tourism, serves individuals seeking preventive care, relaxation, and medical treatments in diverse global destinations. This bibliometric study leverages Scopus for bibliographic data to analyze the scientific production in health tourism. The analysis, conducted using Biblioshiny and CiteSpace, focuses on annual scientific production, identifies the most productive authors, and highlights the most relevant sources. Additionally, the study examines countries’ scientific outputs and provides a historiographic overview of the field. Trend topics and thematic maps visualize the evolution of research themes, while keywords with the strongest citation bursts are identified. Co-citation analysis reveals influential works and collaborations, and a timeline view of country collaborations illustrates the global research network. The study concludes that while health tourism research has significantly expanded, there is a need for longitudinal studies on long-term outcomes and patient satisfaction. Furthermore, the integration of wellness and medical services, as well as the exploration of ethical and legal frameworks, remain underdeveloped. Practical implications suggest that policymakers should focus on developing uniform regulations and resilient practices to enhance the sustainability and attractiveness of health tourism. These findings provide a comprehensive overview of the current state and future directions of health tourism research, highlighting critical areas for further investigation.

## Introduction and background

Health tourism is an exciting phenomenon that encompasses both wellness and medical tourism, dealing with dimensions of health [1,2]. Wellness tourism includes physical, mental, emotional, occupational, intellectual, and spiritual health, referenced by activities focused on the prevention and promotion of health through fitness, prevention and promotion of health through nutrition, relaxation, indulgence, and healing treatments. Some of the popular spots are Iceland, Hawaii, Bali, and Thailand [3-5]. In contrast, medical tourism entails traveling in search of evidence-based medical services, either invasive or non-invasive, for diagnosis, treatment, prevention, and rehabilitation [3,6]. All of these activities take place away from home and are driven by the urge to have affordable and accessible medical procedures unavailable in their countries. These popular destinations include Turkey, India, and Thailand. These approaches make tourism very versatile. Health tourism, driven by increasing awareness of health and a priority on well-being through relaxation and medical care, is very appealing [7].

Countries such as India, Thailand, Malaysia, Mexico, and Turkey are more favored due to the blend of good healthcare services and lower costs. Often, these countries use marketing investments directed at their health services to attract a vast number of international patients [8,9]. For many patients, treatments abroad can be significantly cheaper compared to those sought locally, especially for procedures not covered by their home country’s national health insurance or those that involve high out-of-pocket expenses due to limited or no insurance coverage [3]. Besides, some destinations are also gaining popularity for offering specific kinds of treatments or therapies that are cutting-edge or difficult to access in a patient’s home country. These include state-of-the-art surgeries, alternative medicine, and holistic treatments [8,10]. Mexico has become a popular destination for medical tourists from the United States and Canada, offering affordable treatments, especially in dental care, cosmetic surgery, and bariatric surgery. The country’s proximity to North America, combined with its robust healthcare infrastructure, makes it an attractive option for patients seeking cost-effective treatments. Portugal, on the other hand, is gaining recognition for its wellness tourism, particularly in spa treatments and rehabilitation services. The country’s scenic landscapes, mild climate, and state-of-the-art facilities provide an ideal setting for patients seeking recovery and relaxation. Both countries have strategically invested in marketing their health services to attract a vast number of international patients, leveraging their unique strengths in the health tourism market.

Health tourism offers numerous benefits, including the promotion, stabilization, and restoration of physical, mental, and emotional health, which enhances overall life quality [11]. From an economic perspective, it is highly contributing to the development of countries because it provides alternative treatments, creates business activity, generates wealth, and provides employment opportunities, and thus is among the key sectors of the world economy [12]. Wellness tourism, in particular, aims to improve the quality of life through physically and psychologically healthier citizens with a cleaner environment [13]. Other than this, medical tourism provides private medical services at reasonable costs, thereby assisting patients in getting the latest treatments at relatively cheaper rates than their countries of origin [7]. For purposes of safety and security, governments and institutions need to confirm that health services are indeed factual to ensure a secure environment for health tourists [14].

The challenges of health tourism are multifaceted, involving diverse services and stakeholders, economic and policy issues, and sustainability concerns. In terms of services, contributions by private health units are enormous in areas such as dialysis, orthopedics, oncology, and gynecology [15]. Moreover, this also requires the integration of healthcare providers, government entities, and the tourism sector [16]. Critical challenges in economic and policy terms include how high-quality services can be offered at low costs, how touristic and cultural attractions might be exploited to maximum advantage, and how the climatological conditions might be used best to attract tourists [15]. More broadly, governments seek to foster economic growth while curtailing expenditure on public healthcare. Local perceptions of well-being are, however, often subordinated to the dictates of economics, which complicates sharing benefits equitably [17]. Hence, sustainable development in health tourism is supposed to cope with these environmental, social, and economic challenges [18]. Countries like India seek to balance their traditional and modern healthcare advantages against the hurdles to establish themselves as global healthcare destinations [2].

Bibliometric analysis is a valuable tool, applying the power of quantitative techniques to the academic literature to explain the situation in a specific realm of research [19,20]. This methodology identifies critical areas of research and influential studies, as well as emerging trends, by measures such as publication counts and citation patterns, including authorship networks [21,22]. Such an approach to health tourism would, therefore, be able to trace the evolution of research back to its path, underline the most prolific contributors, and identify the links between the various research themes.

The research questions for the bibliometric study on health tourism aim to explore several critical aspects of the field. What are the primary publication trends in health tourism research, and how have these trends evolved over time? Which authors and journals have had the most significant impact on the field of health tourism, and what are their main contributions? What are the key themes and topics that have emerged in health tourism research, and how have these themes evolved over the decades? What are the main geographical regions contributing to health tourism research, and how do collaboration patterns vary across different countries? Finally, what are the undiscovered research areas in the realm of health tourism research, and what implications do they have for future studies? These questions collectively aim to provide a comprehensive understanding of the development, influence, and future directions of health tourism research.

## Review

Methodology

To conduct a robust bibliometric analysis of health tourism, we employed advanced software tools such as Biblioshiny and CiteSpace. Biblioshiny, an open-source web application with a graphical user interface, facilitates bibliometric analysis and visualization within R, enabling users to explore publication data, perform descriptive analyses, and create scientific landscapes [23,24]. CiteSpace is another powerful tool used for visualizing and analyzing trends and patterns in scientific literature [25,26]. For this study, we utilized the Scopus database due to its comprehensiveness and wide coverage of peer-reviewed literature across disciplines [27]. Using the keywords “health tourism” OR “medical tourism” OR “wellness tourism,” we retrieved publications without any language restrictions, gathering 3,397 documents from 1,559 different sources spanning the years 1963 to 2024.

**Figure 1:**
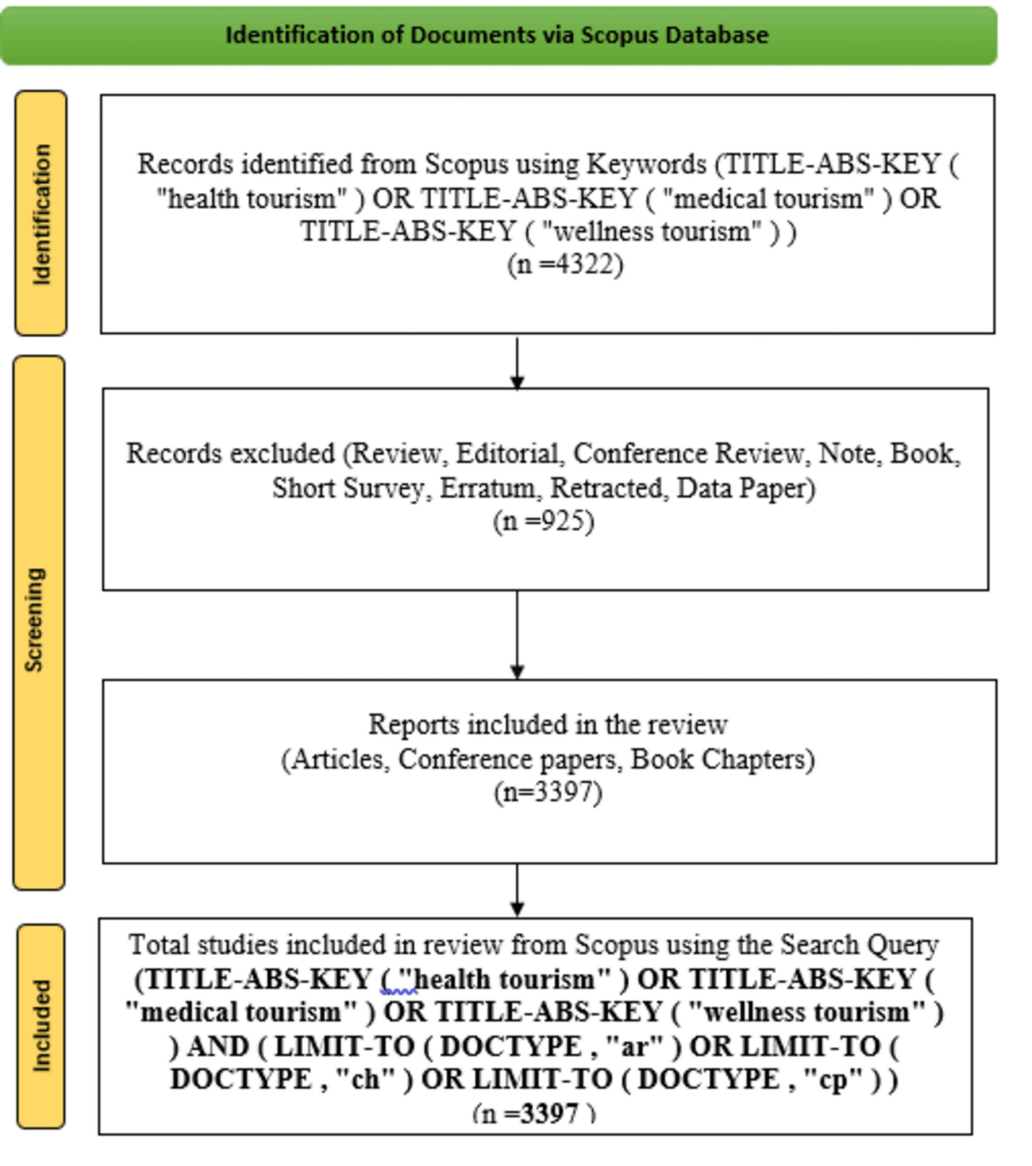
Preferred Reporting Items for Systematic Reviews and Meta-Analyses (PRISMA) flowchart used to select papers for the analysis.

We followed the Preferred Reporting Items for Systematic Reviews and Meta-Analyses (PRISMA) approach, a three-phase procedure, to select papers for bibliometric analysis. In the first phase, we identified and extracted data from the databases. The second phase involved excluding reviews, editorials, books, short notes, surveys, errata, and retracted articles, retaining only articles, conference papers, and book chapters. The data was saved as a CSV file for further analysis. Figure 1 illustrates the PRISMA approach used in this selection process. Subsequent analysis was performed using Biblioshiny and CiteSpace to examine annual scientific production, identify the most productive authors, and explore the sources related to our areas of interest. Our scrutiny of scientific publications worldwide allowed us to establish a historical record of what has been produced over time. Additionally, we presented trending topics through a thematic map, analyzed keywords with the strongest citation burst, examined co-cited literature, and visualized country collaboration on a timeline. These analyses provided valuable insights into shifting focus areas and emerging themes in health tourism research, as well as key publications shaping the field and global authorship collaboration.

Primary Information of the Investigation

Table 1 provides a comprehensive overview of the bibliometric analysis of health tourism. The analysis spanned from 1963 to 2024, including a wide range of sources such as journals, books, and other types, totaling 1,559 sources. A total of 3,397 documents were analyzed, with an annual growth rate of 8.68%. These documents were, on average, 6.82 years old, indicating that the research is up-to-date. The average number of citations per document was 14.02 since the start, with a total of 113,311 references across all documents. The documents contained 8,438 Keywords Plus (ID) and 6,805 Author’s Keywords (DE), subsequently representing the breadth of subjects covered within health tourism research. This is based on an analysis of 8,196 authors (697 with single-authored documents). Of these documents, 814 were single-authored, and the average number of co-authors per document was lower at 3.06, indicating a collaborative research environment. Few were international co-authorships (17.9% of documents), reflecting the fact that health tourism research is a global effort. Concerning document types, the analysis involved 2,610 articles and 486 book chapters and conference papers (presenting a wide variety of publication types across fields). The data offer significant accounts of transformations from birth and shaping the health tourism research landscape, highlighting its trends in growth related to collaboration patterns or publication type diversities.

**Table 1:** Primary information of the investigation.

Description	Results
Main information about the data	
Timespan	1963–2024
Sources (Journals, books, etc.)	1,559
Documents	3,397
Annual growth rate %	8.68
Document average age	6.82
Average citations per doc	14.02
References	113,311
Document contents	
Keywords Plus (ID)	8,438
Author’s Keywords (DE)	6,805
Authors	
Authors	8,196
Authors of single-authored docs	697
Authors’ collaboration	
Single-authored docs	814
Co-Authors per Doc	3.06
International co-authorships %	17.9
Document types	
Article	2,610
Book chapter	486
Conference paper	301

Results

Annual Scientific Production

**Figure 2:**
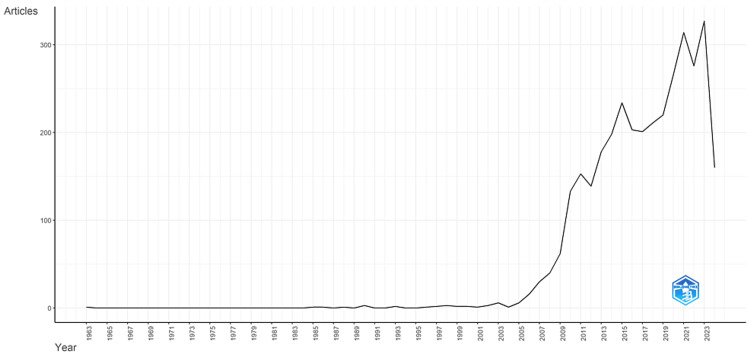
Publications trends in health tourism from 1963 to 2024. Biblioshiny: https://www.bibliometrix.org/home/index.php/layout/biblioshiny.

Figure 2 shows the overview of all publications on health tourism published from 1963 to 2024. Distinct trends are draftable across these decades. For example, the topic in its early years, 1963-1999, was minimally researched. Publications were few and far between, and there were many years of no activity. Remarkable years for publications were 1963, 1985, 1986, and 1988. From 2000 to 2009, there was a visible increase in articles, especially in 2006, with 16 and dramatic increases by 2009 to 62, which marked a rise in academic interest. Since 2010, there has been a rapid and continuously increasing trajectory of publications, peaking up to 327 articles in 2023 and 160 articles as of June 2024. The health tourism trend rapidly acquires prime importance in the academic and research fraternity due to globalization, improved health trajectories, and growing wellness and medical tourism interests.

Most Relevant Authors

Table 2 presents some of the most relevant authors in health tourism research and brings out very insightful data about who has been leading contributions to the knowledge base. In this case, Crooks VA emerged as the most prolific author with 48 publications, denoting strong and sustained interest in health tourism, placing them at the center of the academic discourses. Snyder J follows closely in the second position with 47 publications, marking considerable influence and engagement in increasing inquiry and wisdom accumulation. Further, the critical contributor, Johnston R, has 29 publications, which suggests deep engagement and broad impact on the field. Medhekar A has 19 publications; his work played a significant role in shaping an understanding and developing health tourism research. Next is Ormond M, with 18 publications, making him very active and contributing to the diversity and depth of the area under study. Lunt N has 16 publications that position him as an essential voice, adding valuable insights into the discussion. Horsfall D, with 15 publications, enriches the academic landscape, while both Lee TJ and Turner l feature 14 publications, each of considerable value and breadth. Adams K has 13 publications to make a valuable contribution that underlines their role in developing health tourism research. All authors listed in this section have contributed extensive, impactful publications to help shape discourse, identify trends, and point out critical issues related to health tourism.

**Table 2:** Most relevant authors.

Author	Number of documents
Crooks VA	48
Snyder J	47
Johnston R	29
Medhekar A	19
Ormond M	18
Lunt N	16
Horsfall D	15
Lee TJ	14
Turner L	14
Adams K	13

Most Relevant Sources

Table 3 lists the most impactful journals in the field of health tourism based on the number of articles published. Leading the list is Sustainability (Switzerland), which has 55 articles, indicating a strong focus on sustainable practices within the health tourism sector. The International Journal of Environmental Research and Public Health follows with 41 articles showing significant engagement with environmental and public health perspectives in health tourism. The International Journal of Spa and Wellness and Tourism Management has published 34 articles each, highlighting their roles in wellness tourism and management practices. The Geojournal of Tourism and Geosites, with 33 articles, contributes extensively to geographical and site-specific tourism research. Social Science and Medicine, with 31 articles, integrates social science perspectives with medical tourism. The Journal of Travel and Tourism Marketing, with 30 articles, emphasizes the marketing aspects of travel and health tourism. Springer Proceedings in Business and Economics has 29 articles focusing on health tourism’s business and economic dimensions. The Asia Pacific Journal of Tourism Research and the International Journal of Tourism Research have 24 articles each, contributing to regional and emerging trends in the field.

**Table 3:** Most relevant sources.

Sources	ISSN	Publisher	Country	Articles
Sustainability (Switzerland)	2071-1050	MDPI	Switzerland	55
International Journal of Environmental Research and Public Health	1660-4601	MDPI	Switzerland	41
International Journal of Spa and Wellness	2472-1743	Routledge	United States	34
Tourism Management	0261-5177	Elsevier Ltd.	United Kingdom	34
Geojournal of Tourism and Geosites	2065-0817	Editura Universitatii din Oradea	Romania	33
Social Science and Medicine	0277-9536	Elsevier Ltd.	United Kingdom	31
Journal of Travel and Tourism Marketing	1054-8408	Routledge	United States	30
Springer Proceedings in Business and Economics	2198-7246	Springer Nature	Switzerland	29
Asia Pacific Journal of Tourism Research	1094-1665	Taylor and Francis Ltd.	United Kingdom	24
International Journal of Tourism Research	1099-2340	John Wiley and Sons Ltd.	United Kingdom	24

Countries’ Scientific Productions

Table 4 presents the scientific production in health tourism by country, highlighting the leading contributors in the field. The United States tops the list with 1,400 documents, demonstrating its dominant role and extensive research output in health tourism. China follows with 781 documents, indicating significant research activity and interest in the field. India is third with 636 documents, showcasing its growing contribution to health tourism research. The United Kingdom, with 529 documents, and Malaysia, with 484 documents, are also notable contributors, reflecting their active research communities. Canada (428 documents) and Iran (392 documents) further illustrate the global interest and scholarly contributions to health tourism. Australia (315 documents), South Korea (313 documents), and Thailand (307 documents) round out the list, emphasizing the international scope and diverse geographical interest in health tourism research. These figures underscore the global engagement and the collaborative efforts of various countries in advancing the field of health tourism.

**Table 4:** Countries’ scientific productions.

Region	Number of documents
United States	1,400
China	781
India	636
United Kingdom	529
Malaysia	484
Canada	428
Iran	392
Australia	315
South Korea	313
Thailand	307

Historiograph

**Figure 3:**
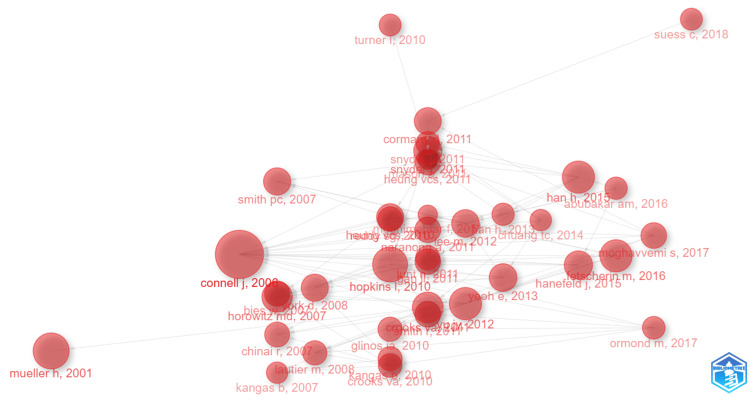
Evolution and connections of the most influential publications. Biblioshiny: https://www.bibliometrix.org/home/index.php/layout/biblioshiny.

The historiograph depicted in Figure 3 is a visualization tool that plots the evolution and connections of the most influential publications in health tourism research. It underlines key elements and thematic evolvement over time. Key publications, such as Connell J 2006 and Mueller HR 2001, appear as larger nodes, thereby portraying their primary nature with a high citation count. Probably, Connell’s contribution laid the ground for essential concepts or frameworks that most of the subsequent studies have built on. The early research by Mueller probably laid the groundwork for further field development. Prominently connected influential authors include Smith PC 2007 and Turner L 2010, whose research was highly cited, hence instrumental in shaping subsequent research publications. Influential publications include those by Han HH 2015, Abubakar AM 2016, and Heung VCS. Moreover, they are located in central and well-connected positions in 2011, which means that these studies contributed much toward pushing forward the health tourism research in the mid-2010s. This historiography strongly marks the different phases of the research focus by having well-defined clusters of topics. The earlier research from 2001 to 2006, represented by Mueller and Connell, addressed definitions in the early stages, economic implications, and early case studies related to health tourism. A marked increase of highly interconnected nodes exists in the 2007-2013 period, showing topic diversification. During this period, critical studies were contributed by Smith, Connell, and Turner, who examined several issues about medical tourism, wellness tourism, and perceptions of patients. Recent development: 2014-2018; authors Han, Abubakar, Ormond; issues explored on ethical considerations, global health, and patient decision processes in contemporary times. This progression highlights the field’s dynamic nature, moving from foundational concepts to more specialized and diversified themes. The historiograph effectively demonstrates how early foundational studies influenced subsequent research, showcasing the cumulative nature of knowledge development in health tourism.

Trend Topics

**Figure 4:**
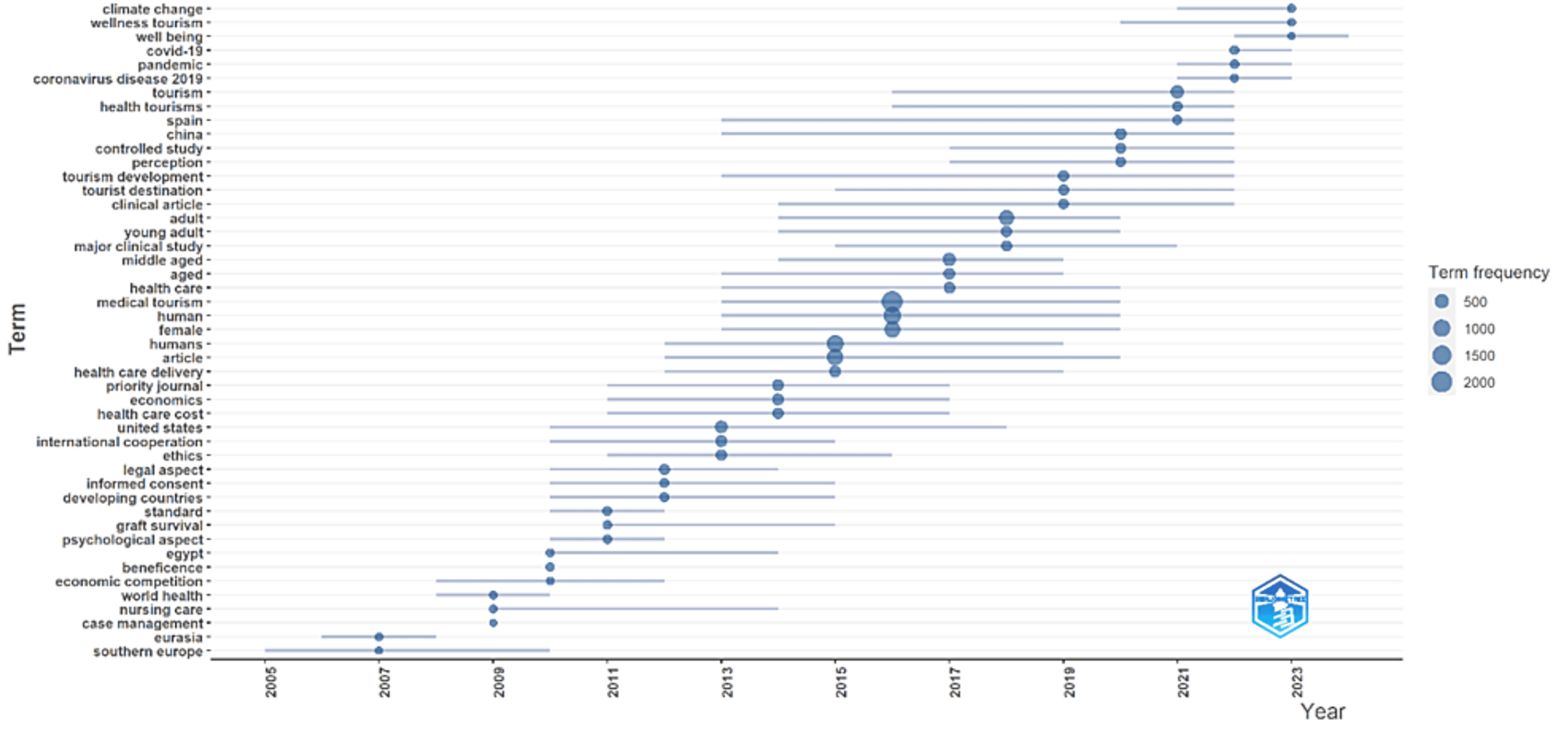
Trending topics over the past 20 years. Biblioshiny: https://www.bibliometrix.org/home/index.php/layout/biblioshiny.

Figure 4 illustrates the trending topics over the past 20 years, providing valuable insights into evolving focus areas and emerging themes. From 2005 to 2010, early research topics included “case management,” “nursing care,” “graft survival,” and “economic competition,” indicating a focus on the practical and economic aspects of health tourism, such as the management of healthcare services and economic implications. Terms such as “southern Europe” and “Eurasia” pointed to a geographical interest, while “beneficence” and “ethics” reflected emerging concerns with ethical issues. Between 2010 and 2015, the scope of research broadened significantly to include “health care delivery,” “medical tourism,” “developing countries,” “psychological aspect,” and “legal aspect.” This period saw an increased emphasis on the delivery systems, psychological impacts, legal considerations, and the necessity for international cooperation, as evidenced by the prominence of “international cooperation.” There was also a deeper exploration of ethical implications and financial aspects, marked by an increased focus on “ethics,” “informed consent,” and “health care cost.” From 2015 to 2020, research topics shifted toward a clinical and healthcare focus, with terms such as “clinical article,” “tourist destination,” “healthcare,” “middle aged,” and “young adult” indicating a strong focus on clinical studies, specific demographics, and the role of tourist destinations in health tourism. The emergence of “perception” and “tourism development” suggested an interest in understanding how health tourism is perceived and how it can be effectively developed. Significant research efforts were highlighted by the appearance of “major clinical study.” The years 2020 to 2024 were heavily influenced by the impact of the COVID-19 pandemic, with terms such as “coronavirus disease 2019,” “COVID-19,” and “pandemic” reflecting this profound impact. Emerging topics such as “climate change,” “wellness tourism,” “well being,” and “sustainability” indicated a shift toward sustainable practices and a holistic approach to health tourism. The focus on specific geographical areas and the overarching management and organization of health tourism were evident in terms such as “China,” “Spain,” “tourism,” “health tourism,” and “tourism management.” These trends illustrate a dynamic field that has expanded from practical and economic aspects to include ethical, psychological, legal, and global dimensions, showcasing its adaptability and comprehensive approach to current global challenges and trends.

Thematic Map

**Figure 5:**
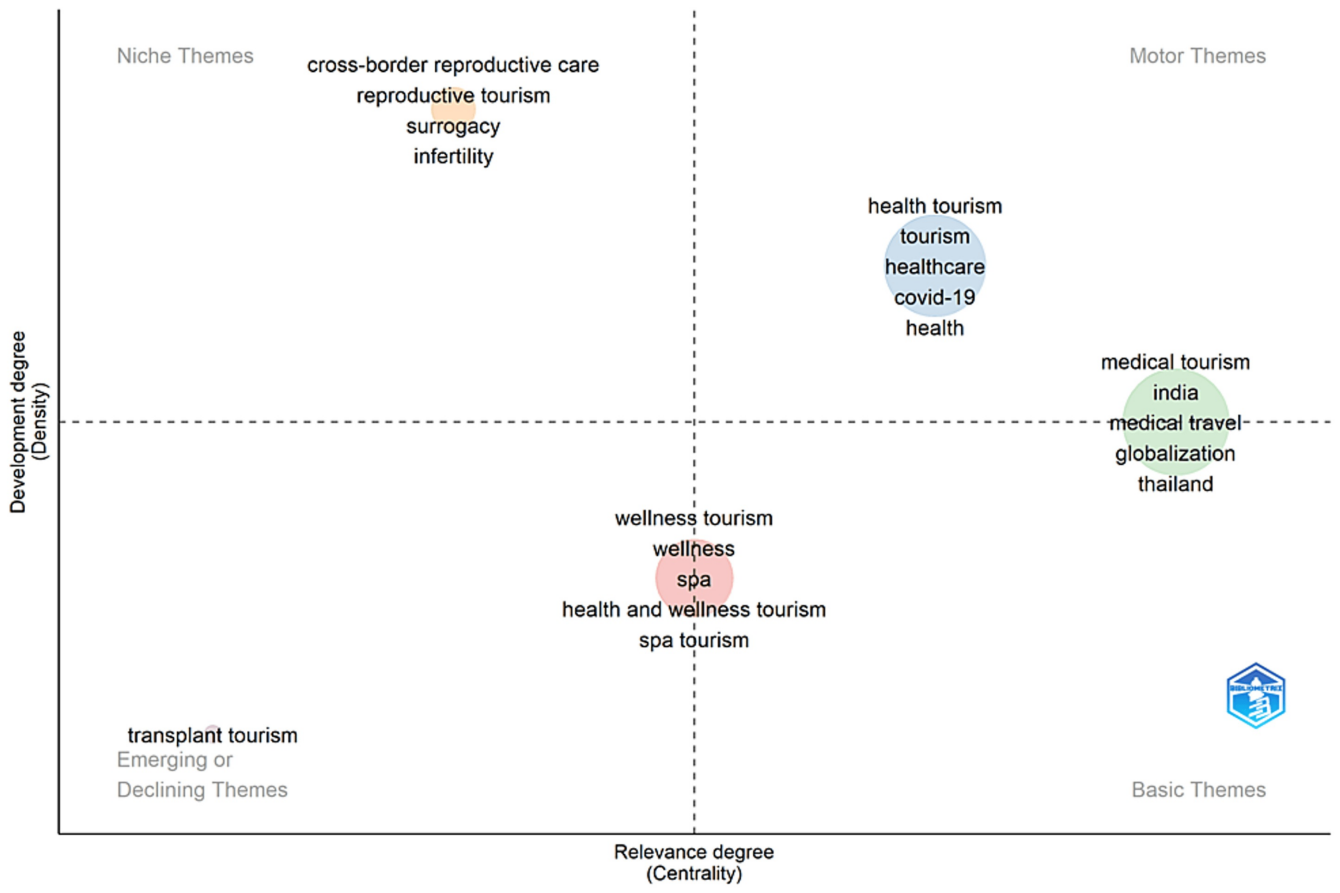
Themes grouped by their level of development and significance. Biblioshiny: https://www.bibliometrix.org/home/index.php/layout/biblioshiny.

The thematic map shown in Figure 5 illustrates the stage of development and the significance of different themes based on two dimensions: Development level (Density) and Relevance level (Centrality). Motor themes, such as health tourism and medical tourism, are well-developed and central to the research field, indicating they are crucial and have strong internal development. Niche themes, such as cross-border reproductive care, are specialized and well-developed internally but are less central to the overall research field. Basic themes are important foundational aspects of the field. Still, they are not highly developed, while emerging or declining themes, such as transplant tourism, have low centrality and density, indicating they are either emerging or possibly losing relevance. Wellness tourism appears close to the center of the map, indicating it is a balanced theme with moderate development and relevance. This theme includes keywords such as wellness, spa, health and wellness tourism, and spa tourism. Health tourism and medical tourism are highly developed and central to the field, making them the key focus areas in health tourism research. In contrast, cross-border reproductive care is a specialized niche with substantial internal development but less connection to other themes in health tourism. Transplant tourism is an emerging or possibly declining area, indicating it requires more research or might be losing relevance. Overall, this map helps researchers and practitioners understand which areas are well-studied and crucial and which might need more focus or could be emerging trends.

Keywords With the Strongest Citation Bursts

Table 5 presents the top 25 keywords with the most significant citation bursts from 2010 to 2024. Citation bursts indicate periods when specific keywords received a surge in citations, reflecting heightened interest and relevance in the research community. Early in the decade, keywords such as “legal aspect” (2010-2014), “psychological aspect” (2010-2013), “ethics” (2010-2013), and “statistics” (2010-2014) had significant bursts. These keywords highlight the early focus on legal considerations, psychological dimensions, ethical issues, and statistical analyses, which laid the groundwork for more complex discussions in health tourism. Research in the mid-2010s saw sustained interest in themes such as “international cooperation” (2010-2015), “health care quality” (2013-2016), and “statistics and numerical data” (2013-2018), indicating a broader and more collaborative approach to addressing health tourism challenges, with an emphasis on maintaining and improving healthcare standards. In the late 2010s to early 2020s, there was a noticeable shift toward sustainability and the impacts of the COVID-19 pandemic. Keywords such as “trends” (2014-2018), “sustainable development” (2020-2024), and “sustainability” (2020-2024) reflect a growing emphasis on sustainable practices within health tourism. The keyword “COVID-19” (2021-2024) had the highest burst strength of 21.36, underscoring the pandemic’s significant impact on health tourism research. Currently, there is a strong focus on keywords such as “tourism” (2020-2024), “health tourisms” (2012-2024), “pandemic” (2020-2024), “wellness tourism” (2021-2024), and “health tourism” (2022-2024). These terms indicate ongoing interest in holistic health, wellness aspects, and the broad impact of pandemics on the sector.

**Table 5:** Top 25 keywords with the most significant citation bursts from 2010 to 2024.

Keywords	Year	Strength	Begin	End	1985–2024
travel	1990	12.41	1990	2009	▂▂▂▂▂▃▃▃▃▃▃▃▃▃▃▃▃▃▃▃▃▃▃▃▃▂▂▂▂▂▂▂▂▂▂▂▂▂▂▂
standard	2007	15.33	2007	2013	▂▂▂▂▂▂▂▂▂▂▂▂▂▂▂▂▂▂▂▂▂▂▃▃▃▃▃▃▃▂▂▂▂▂▂▂▂▂▂▂
internationality	2007	12.07	2007	2013	▂▂▂▂▂▂▂▂▂▂▂▂▂▂▂▂▂▂▂▂▂▂▃▃▃▃▃▃▃▂▂▂▂▂▂▂▂▂▂▂
united states	1990	11.08	2007	2010	▂▂▂▂▂▂▂▂▂▂▂▂▂▂▂▂▂▂▂▂▂▂▃▃▃▃▂▂▂▂▂▂▂▂▂▂▂▂▂▂
legal aspect	2006	23.3	2009	2014	▂▂▂▂▂▂▂▂▂▂▂▂▂▂▂▂▂▂▂▂▂▂▂▂▃▃▃▃▃▃▂▂▂▂▂▂▂▂▂▂
ethics	2009	11.66	2009	2013	▂▂▂▂▂▂▂▂▂▂▂▂▂▂▂▂▂▂▂▂▂▂▂▂▃▃▃▃▃▂▂▂▂▂▂▂▂▂▂▂
psychological aspect	2010	14.64	2010	2013	▂▂▂▂▂▂▂▂▂▂▂▂▂▂▂▂▂▂▂▂▂▂▂▂▂▃▃▃▃▂▂▂▂▂▂▂▂▂▂▂
statistics	2010	10.52	2010	2014	▂▂▂▂▂▂▂▂▂▂▂▂▂▂▂▂▂▂▂▂▂▂▂▂▂▃▃▃▃▃▂▂▂▂▂▂▂▂▂▂
organ transplantation	2010	9.78	2010	2013	▂▂▂▂▂▂▂▂▂▂▂▂▂▂▂▂▂▂▂▂▂▂▂▂▂▃▃▃▃▂▂▂▂▂▂▂▂▂▂▂
health care quality	2007	11.02	2013	2016	▂▂▂▂▂▂▂▂▂▂▂▂▂▂▂▂▂▂▂▂▂▂▂▂▂▂▂▂▃▃▃▃▂▂▂▂▂▂▂▂
statistics and numerical data	2013	22.99	2014	2018	▂▂▂▂▂▂▂▂▂▂▂▂▂▂▂▂▂▂▂▂▂▂▂▂▂▂▂▂▂▃▃▃▃▃▂▂▂▂▂▂
trends	2014	16.46	2014	2018	▂▂▂▂▂▂▂▂▂▂▂▂▂▂▂▂▂▂▂▂▂▂▂▂▂▂▂▂▂▃▃▃▃▃▂▂▂▂▂▂
legislation and jurisprudence	2013	12.18	2014	2018	▂▂▂▂▂▂▂▂▂▂▂▂▂▂▂▂▂▂▂▂▂▂▂▂▂▂▂▂▂▃▃▃▃▃▂▂▂▂▂▂
procedures	2014	10.93	2014	2019	▂▂▂▂▂▂▂▂▂▂▂▂▂▂▂▂▂▂▂▂▂▂▂▂▂▂▂▂▂▃▃▃▃▃▃▂▂▂▂▂
standards	2015	11.8	2015	2018	▂▂▂▂▂▂▂▂▂▂▂▂▂▂▂▂▂▂▂▂▂▂▂▂▂▂▂▂▂▂▃▃▃▃▂▂▂▂▂▂
human experiment	2016	11.14	2017	2024	▂▂▂▂▂▂▂▂▂▂▂▂▂▂▂▂▂▂▂▂▂▂▂▂▂▂▂▂▂▂▂▂▃▃▃▃▃▃▃▃
tourist destination	2011	9.54	2019	2024	▂▂▂▂▂▂▂▂▂▂▂▂▂▂▂▂▂▂▂▂▂▂▂▂▂▂▂▂▂▂▂▂▂▂▃▃▃▃▃▃
tourism	1993	34.81	2020	2024	▂▂▂▂▂▂▂▂▂▂▂▂▂▂▂▂▂▂▂▂▂▂▂▂▂▂▂▂▂▂▂▂▂▂▂▃▃▃▃▃
sustainable development	2007	11.99	2020	2024	▂▂▂▂▂▂▂▂▂▂▂▂▂▂▂▂▂▂▂▂▂▂▂▂▂▂▂▂▂▂▂▂▂▂▂▃▃▃▃▃
covid 19	2021	21.82	2021	2024	▂▂▂▂▂▂▂▂▂▂▂▂▂▂▂▂▂▂▂▂▂▂▂▂▂▂▂▂▂▂▂▂▂▂▂▂▃▃▃▃
pandemic	2020	12.6	2021	2024	▂▂▂▂▂▂▂▂▂▂▂▂▂▂▂▂▂▂▂▂▂▂▂▂▂▂▂▂▂▂▂▂▂▂▂▂▃▃▃▃
health tourisms	2012	11.11	2021	2024	▂▂▂▂▂▂▂▂▂▂▂▂▂▂▂▂▂▂▂▂▂▂▂▂▂▂▂▂▂▂▂▂▂▂▂▂▃▃▃▃
wellness tourism	2010	36.05	2022	2024	▂▂▂▂▂▂▂▂▂▂▂▂▂▂▂▂▂▂▂▂▂▂▂▂▂▂▂▂▂▂▂▂▂▂▂▂▂▃▃▃
health tourism	1993	12.59	2022	2024	▂▂▂▂▂▂▂▂▂▂▂▂▂▂▂▂▂▂▂▂▂▂▂▂▂▂▂▂▂▂▂▂▂▂▂▂▂▃▃▃
china	2009	9.84	2022	2024	▂▂▂▂▂▂▂▂▂▂▂▂▂▂▂▂▂▂▂▂▂▂▂▂▂▂▂▂▂▂▂▂▂▂▂▂▂▃▃▃

Network Visualization of Co-citation of Cited Authors

**Figure 6:**
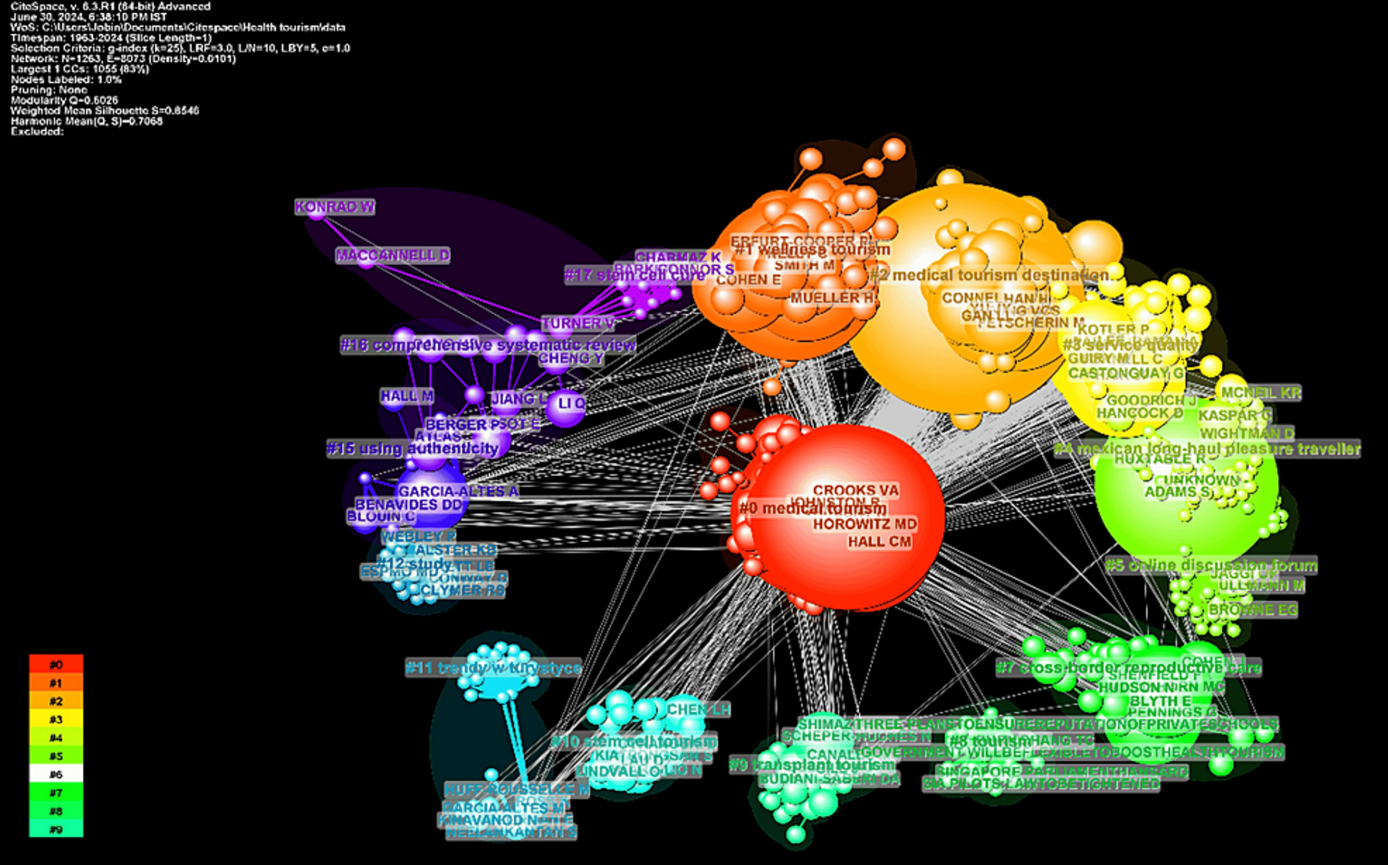
Co-citation of cited authors. CiteSpace: https://citespace.podia.com/.

The visualization in Figure 6 illustrates the interconnectedness and thematic organization of influential works through the co-citation of cited authors. The network consists of 15 clusters, each representing a distinct area of focus within the field. Cluster #0, labeled as “medical tourism,” is the largest, with 210 members and a silhouette value of 0.773, indicating it is a well-defined group with substantial research activity. Key contributors to this cluster include Lunt N, Turner L, and Crooks VA, whose work has significantly shaped the field. Cluster #1, “wellness tourism,” with 193 members and a silhouette value of 0.783, also highlights important aspects of health tourism, with major contributions from Smith M, Voigt C, and Mueller H. Cluster #2, “medical tourism destination,” has 148 members and is another central theme, underscored by the influential works of Connell J, Han H, and Heung VCS. Other notable clusters include Cluster #3, also focused on “medical tourism,” with major contributions from Hair Jf, Fornell C, and Parasuraman A. Cluster #4, “Mexican long-haul pleasure traveller,” and Cluster #5, “online discussion forum,” indicate niche areas of research with high silhouette values, suggesting well-defined topics within their scope. Clusters like #7, “cross-border reproductive care,” and #9, “transplant tourism,” highlight the ethical and legal implications of health tourism. The diversity of clusters, ranging from “stem cell tourism” in Cluster #10 to “using authenticity” for competitive advantage in Cluster #15, showcases the breadth of research areas. The presence of comprehensive systematic reviews in Cluster #16 and the emerging focus on “stem cell cure” in Cluster #17 further illustrate the dynamic and evolving nature of health tourism research. This visualization helps identify key research areas and influential works, offers a comprehensive overview of the health tourism research landscape, and guides future research directions.

*Timeline Network Visualization of Countries*’* Collaborations*

**Figure 7:**
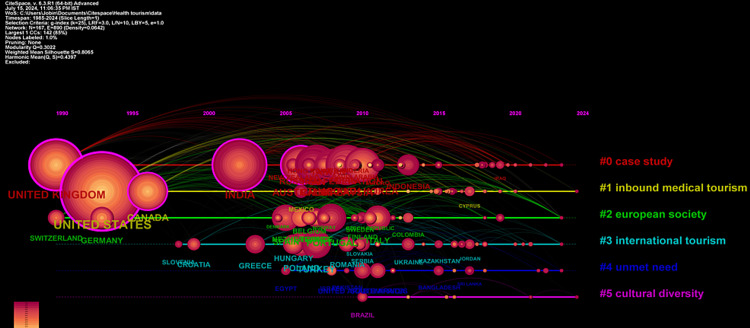
Network visualization of countries’ collaborations. CiteSpace: https://citespace.podia.com/.

The network visualization in Figure 7 illustrates country collaborations, revealing six major clusters of interconnected and collaborative efforts across nations. The largest cluster, Cluster #0, labeled as “Case Study,” comprises 44 members and focuses on medical tourism and cross-border healthcare directives. Significant contributions from India (286 citations), the United Kingdom (249 citations), and Australia (187 citations) underscore the importance of these countries in researching healthcare-seeking behaviors during travel. Similarly, Cluster #1, “Inbound Medical Tourism,” with 26 members, emphasizes the leading role of the United States (564 citations) in inbound medical tourism research, followed by Canada (142 citations) and Mexico (25 citations). This cluster highlights North America’s substantial contributions to understanding the dynamics of patients traveling to receive medical care. Cluster #2, “European Society,” comprising 25 members, underscores collaborative efforts within Europe, particularly on cross-border healthcare, with notable contributions from Portugal (92 citations), Spain (90 citations), and Italy (62 citations). Cluster #3, “International Tourism,” with 24 members, focuses on sustainable tourism practices post-COVID-19, with key contributions from Turkey (108 citations), Poland (76 citations), and Hungary (54 citations). Cluster #4, “Unmet Need,” comprising 15 members, addresses ethical and legal aspects of medical tourism, with significant contributions from the United Arab Emirates (38 citations), South Africa (37 citations), and Israel (25 citations). Lastly, Cluster #5, “Cultural Diversity,” with eight members, highlights the importance of respecting cultural differences in medical tourism, with Brazil (22 citations) being a significant contributor. Overall, this visualization provides a comprehensive overview of country collaborations in health tourism research, highlighting key players and thematic focuses across different regions, and offering insights into the global research landscape in health tourism.

Discussion

The historical context of health tourism research reveals a field that has grown substantially over the past decades. Initially, the field saw minimal activity from 1963 to 1999, a period characterized by sporadic publications and limited academic interest. However, the turn of the millennium marked a significant shift, with a notable increase in publications starting in 2000 and peaking in 2009 [28]. This surge can be attributed to several factors, including the 2008 Global Financial Crisis, which likely heightened interest in cost-effective healthcare options abroad, driving both academic and industry focus on medical tourism [29]. The continuous rise in publications post-2010 reflects the growing importance of health tourism in the context of globalization, advancements in medical technology, and the increasing accessibility of international travel.

The impact of global events, particularly the COVID-19 pandemic, has further shaped the research landscape in health tourism. The pandemic has underscored the importance of sustainability and resilience in health tourism practices, leading to a shift in research priorities [30]. For example, recent studies have focused on the role of telemedicine as an alternative to international travel for medical procedures, the resilience of health tourism businesses during global health crises, and the growing emphasis on local wellness tourism as a safer alternative during periods of travel restrictions. These shifts highlight the dynamic nature of health tourism research and its ability to adapt to global challenges. This study does not attempt to reproduce their complete citation networks, rather it focuses on identifying points of intellectual trajectory and topics as they begin a conversation in literature. This study enhances knowledge of discourse analysis for health tourism research by offering an extensive review, playing a guiding role in future studies, and ascertaining the basis on which policy-making decisions may be arrived at using current literature. These findings highlight the dynamic nature of health tourism research, evolving from foundational studies to more complex themes encompassing ethical, psychological, and global dimensions.

Identifying research gaps provides a roadmap for future studies in health tourism. The absence of longitudinal studies examining the long-term effects of health tourism on patients and healthcare systems represents a significant gap in the literature. Moreover, while psychological well-being post-treatment has been acknowledged as important, there is a need for more in-depth exploration of this area. Additionally, the integration of wellness and medical services remains under-researched, despite its potential to offer a more holistic approach to health tourism.

In addressing these gaps, future research could draw inspiration from successful interdisciplinary studies and policy initiatives. For instance, the integration of wellness and medical services in Thailand, where wellness resorts collaborate with local hospitals, offers a model for other regions seeking to enhance their health tourism offerings. Similarly, the development of uniform regulations for medical tourism within the European Union could serve as a blueprint for establishing ethical and practical standards globally. These examples underscore the importance of interdisciplinary collaboration and policy development in advancing the field of health tourism.

Research gaps and practical implications

The trend topics analysis and the Thematic map in health tourism research during the past 20 years expose several gaps. First, although the early scientific works were focused on the practical and economic themes of health tourism, such as case management and economic competition, longitudinal studies are strikingly absent on long-term effects and consequences for patients and healthcare systems alike. Second, though the period of 2010-2015 was characterized by strengthened attention to the psychological and legal problems of health tourism, there is a need for further studies examining psychological well-being and satisfaction among patients after treatment. Third, the recent focus on studies in the clinic and specific demographics has not been complemented by comprehensive research into the integration of wellness and medical services that offer a holistic approach to health tourism. Although the impact of the COVID-19 pandemic has been well documented, little research examines the long-term implications that the pandemic will have on health tourism and how this may relate to sustainability and climate change.

The findings from the bibliometric analysis provide several practical implications for the future of health tourism. First, further studies with a longitudinal approach are required to theoretically assess the long-term outcomes and patient satisfaction, which can also inform better practices and policies in health tourism. Second, interdisciplinary research that puts wellness and medical services together can give a more comprehensive approach toward health tourism and, hence, enhance its attractiveness and effectiveness. Third, given the increased attention toward ethical and legal matters, policymakers would have to attempt drafting some uniform legislation and regulations for allowing the practice to go forward with ethics and safeguarding the rights of patients in the domain of health tourism. Furthermore, the geographical areas pledging a considerable share in health tourism suggest focused marketing and development policies to materialize the potential therein. Finally, the COVID-19 pandemic underlines the need for building resilient health tourism practices to adapt to global health crises and ensure sustainability.

## Conclusions

This bibliometric analysis has highlighted the dynamic growth of health tourism research, revealing key contributors, trends, and global collaborations. The rapid increase in publications, particularly in the last decade, underscores the field’s evolving nature, driven by globalization, advancements in medical technology, and rising interest in wellness and medical tourism. However, significant research gaps persist, particularly in understanding long-term outcomes and patient satisfaction. Future research should adopt longitudinal studies and interdisciplinary approaches to explore the integration of wellness and medical services using frameworks such as integrative healthcare models to enhance patient care and experience.

Looking forward, the future of health tourism research should focus on addressing the challenges posed by global health crises and developing sustainable practices. There is a pressing need for uniform guidelines and benchmarks across the sector, particularly in regions such as Mexico and Portugal, where health tourism is rapidly expanding. By embracing a more integrative and sustainable approach, and exploring emerging trends such as personalized and digital health services, the field can continue to evolve and make meaningful contributions to global health and well-being.
